# Which urine marker test provides more diagnostic value in conjunction with standard cytology- ImmunoCyt/uCyt+ or Cytokeratin 20 expression

**DOI:** 10.1186/1746-1596-4-20

**Published:** 2009-06-26

**Authors:** Isin Soyuer, Mustafa Sofikerim, Fatma Tokat, Serdar Soyuer, Figen Ozturk

**Affiliations:** 1 Pathology Department, Erciyes University, Medical Faculty, Kayseri, Turkey; 2 Urology Department, Erciyes University, Medical Faculty, Kayseri, Turkey; 3 Radiation Oncology Department, Erciyes University, Medical Faculty, Kayseri, Turkey

## Abstract

**Background:**

Because of the poor sensitivity of urinary cytological findings for the diagnosis of especially low grade urinary bladder carcinoma, new molecular diagnostic methods have been proposed. We decided to verify the ImmunoCyt/uCyt+ (UCyt+™) test and cytology combination and cytokeratin 20 (CK20) and cytology combination in urine as possible diagnostic and monitoring tool for bladder cancer.

**Methods:**

Evaluation of CK20 expression and UCyt+™ was performed in urine of 90 patients of which 54 with bladder cancer with primary/recurrent diagnosis (low grade urothelial carcinoma (LGUC) = 23/8 patients, high grade urothelial carcinoma (HGUC) = 18/5 patients), and 36 patients as control; except of neoplastic bladder disease patients. For the evaluation of the three tests, CK20 and UCyt+™ tests were combined with urine cytology and compared with each other.

**Results:**

The overall sensitivity detected for each tumor marker was as follows: for urine cytology was 75.9% and UCyt+™ was 83.3%, for CK20 70.4%, while the specificity was 66.7% for urine cytology and 86.1% for UCyt+™ and 83.3% for CK20. The sensitivity of cytology and UCyt+™ combination was higher (88.9%) than the sensitivity cytology and CK20 combination (77.8%). The simultaneous use of the three markers, sensitivity was reaching 92.5%.

**Conclusion:**

The UCyt+™ test and CK20 expression are valid tools for the performance of adjunctive analyses with conventional cytologic examination.

## Background

Bladder cancer is the second most common urologic cancer [1]. The majority of patients with newly diagnosed bladder cancers have superficial, low-grade neoplasms that are associated with an excellent prognosis. However, these tumors have a 30% to 70% recurrence rate and may progress to invasive cancers in 10% to 30% of patients; progression greatly increases the risk of metastasis and subsequent mortality [2,3]. For this reason, the early detection of bladder tumors is essential for improved patient prognosis and long-term survival.

Cytology is noninvasive and has high specificity but low sensitivity, especially for low-grade tumors. At the same time, it can be a challenging test to perform and is highly dependent on the skills and experience of a trained cytopathologist. Thus, published studies have reported a wide range of sensitivities (15.8%–84.6%) [4-12].

Because cystoscopy is invasive and because cytology has poor sensitivity, noninvasive biomarkers have been sought as alternatives to cystoscopy and cytology for the detection and surveillance of bladder cancer.

The ImmunoCyt/uCyt+ (UCyt+™) test (Diagnocure) is an immunocytological fluorescence assay designed to improve the sensitivity of cytology. A cocktail of 3 monoclonal antibodies is used to detect antigens originating specifically from tumors of transitional epithelial cells. The M344 and LDQ10 antibodies are labeled with fluorescein, a green fluorescence, and will recognize a mucin-like antigen located in the urine on exfoliated tumor cells. The 19A211 antibody will recognize the presence of a high molecular weight glycosylated form of carcinoembrionic antigen and is labelled with Texas Red [13].

Still, this test, like cytology, remains subjective and depends in part on the technician. Observer experience, specimen stability and handling and differences in sample size may explain the variation in reported UCyt+™ sensitivity [6]. Because of this, we added another marker, cytokeratin 20 (CK 20) which is easily used in conjunction with cytology and comparable with cytology and UCyt+™ test.

Cytokeratins are intermediate filaments expressed in epithelial cells [14]. One of these, cytokeratin 20, is expressed higher in urothelial tumors in comparison with normal transitional epithelium so it can be considered a marker of urothelial differentiation [14].

The aim of this study was to investigate the validity of the UCyt+™ test and CK20 expression alone and in combination with conventional cytology for detecting bladder cancer.

## Methods

This study was performed on 90 patients admitted to the Urology and Pathology Departments, Faculty of Medicine, Erciyes University Hospitals, Turkey. All patients provided a single voided urine sample, and cytological tests of the urine sediment were performed before cystoscopy. Cystoscopy was done for all patients as the reference standard for identification of bladder cancer. All tumors and suspicious lesions found were either resected or biopsied. The final diagnosis of bladder cancer was based on histopathological examination.

To exclude interference with inflammation or hematuria, none of the follow up urine samples was collected earlier than 3 months after TUR-B or 1 month after intravesical instillation.

For the cytologic examination 40 to 100 mL specimens of voided urine were collected. This material was immediately fixed with equal volumes of 50% ethanol and 1 mL of a special fixative solution. Samples were centrifuged at 2500 rpm for 10 minutes. Cytospin preparations 294 mm^2 ^in diameter were prepared on poly-L-lysine-coated slides. Slides were stained by a routine Papanicolaou method and performed microscopic examination. The slides were examined at 40× magnification.

All patients with superficial disease underwent transurethral resection of the bladder. Urothelial cancer grading and staging were performed according to the World Health Organization criteria [15].

On the cytologic examination, specimens were evaluated for adequacy; those with fewer than 10 urothelial cells were designated unsatisfactory and rejected. The primary interpretation for each cytology case was classified as benign or malignant by using previously published cytologic criteria for the diagnosis of carcinoma [16]. Specifically, the cytologic parameters evaluated included increased nuclear size elevated nuclear-to-cytoplasmic (N/C) ratio, nuclear pleomorphism, hyperchromasia, nuclear eccentricity, nuclear membrane irregularity, and cytoplasmic homogeneity. When none or one of these was present, the diagnosis was rendered as "benign". When most or all were present, the case was diagnosed as "malignant". "Atypical or suspicious" cases were added to the malignant group, which had two or three of these parameters but were not sufficient for a "malignant" diagnosis. When calculating the sensitivity, specificity, positive and negative predictive values, the suspicious cases were considered as positive cases.

The UCyt+™ is a commercially available immunocytological assay based upon microscopical detection of tumor-associated cellular antigens in urothelial cells by immunofluorescence (Diagnocure Inc., Quebec, Canada). The test was performed according to the manufacturer's protocol. Voided urine (>30 ml) was prefixed with an equal amount of ethanol (50%) and 0.5 ml fixative solution. The samples were then stored at 4°C for up to 7 days. Slides with less than 500 nuclei or <1 epithelial cell/HPF (200×) were excluded from the study. Positive and negative controls were performed with each test run. The samples were examined at 400× magnification. A sample was considered positive if one or more cells showed red and/or green fluorescence. The test was negative if no red or green fluorescent cells were detected.

Before immunocytochemistry for CK-20 was performed using the standard streptavidin-biotin peroxidase complex method, selected slides were decolorized with 0.5% hydrochloric acid in 95% ethanol. Antigenic epitopes were retrieved by way of a 15-minute incubation with 2% 3-amino-9-ethylcarbazole/1% hydrogen peroxidase/acetate buffer. The slides were then placed in 3% hydrogen peroxide/methanol for 20 minutes to block nonspecific background staining due to endogenous peroxidase activity. The primary antibody CK-20 (clone Ks20.8, Neomarkers) was diluted 1:100 and applied to the slides for 30 minutes, followed by a 20-minute incubation in a secondary antibody (goat anti-mouse Ig) solution. Diaminobenzidine served as the chromagen and Mayer's hematoxylin as the counterstain. The whole procedure was performed at room temperature. An overall 5% of stained cells were set as the threshold for a positive diagnosis.

The immunoreaction was topographically evaluated and scored by the same pathologist. In accordance with previous reports, the CK20 staining pattern was considered normal when an intense reaction was observed in the apical cells, whereas diffuse or absent CK20 immunostaining was considered an abnormal staining pattern.

The analysis of sensitivity, specificity as well as positive and negative predictive values was performed evaluating each test separately and the two and three together. Sensitivity according to tumor grade was carried out for each marker as well as the specificity and they were also evaluated in the control group. Considering the three markers together, the result was considered positive when at least one marker was positively expressed and negative for tumor diagnosis when all the markers were negative.

## Results

Ninety patients included in the study, were diagnosed as either the bladder cancer with primary/recurrent diagnosis or non-neoplastic bladder disease by histopathologic examination. The malignant group consisted of 54 patients (mean age: 66, range 46–80 years) with urothelial carcinoma [LGUC = 31 patients (23 primary/8 recurrent) and, HGUC = 23 patients (18 primary/5 recurrent)]. The control group consisted of 36 patients (mean age: 49, range 30–78 years), which were non-neoplastic bladder disease patients with cystitis (n = 9), glomerular disease (n = 6) and as individuals with benign prostatic hyperplasia (n = 11), or patients with a history of prostate cancer (n = 5) and renal cell carcinoma patients (n = 5).

The primary interpretation for each cytology case was classified as benign (n = 37) or malignant (n = 53). "Atypical or suspicious" cases (n = 14) were added to the malignant group. In control group, of the 36 analyses with no tumor present, cytology was negative in 24 patients and, therefore, the specificity was 66.7% (Table 1). Of the 54 cases in which a tumor was present, cytology were positive in 41 cases, and the sensitivity of cytology was 75.9%. On the other hand; of the 54 cases for which a tumor was present, 45 were positive by UCyt+™ and the sensitivity was 83.3% (Table 1). In the control group, 31 were negative by UCyt+™ and the specificity was 86.1%. Immunocytology with CK20 had a sensitivity of 70.4% and a specificity of 83.3% (Table 1).

**Table 1:** Sensitivity, specificity, and positive (PPV) and negative predictive value (NPV) for cytology, ImmunoCyt/uCyt+, CK20 and combinations.

		**BiOPSY**	**RESULT**			**TEST**	**RESULT**		
		**LGUC** n = 31	**HGUC** n = 23	**Negative**	**Sensitivity**	**Specificity**	**PPV**	**NPV**	**OA**
	
**Cytology**	+	21	20	12	75.9	66.7	77.3	88.9	72.2
	-	10	3	24					
									
**UCyt+™**	+	24	21	5	83.3	86.1	90.0	79.5	84.4
	-	7	2	31					
									
**CK20**	+	20	18	6	70.4	83.3	86.3	65.2	75.5
	-	11	5	30					
									
**Cytology+ UCyt+™**	+	26	22	5	88.9	86.1	90.5	86.1	87.7
	-	5	1	31					
									
**Cytology+ CK20**	+	22	20	12	77.8	66.7	77.7	66.7	73.3
	-	9	3	24					
									
**UCyt+™+ CK20**	+	23	21	7	81.5	80.5	86.3	74.3	81.1
	-	8	2	29					
									
**Cytology+ UCyt+™ + CK20**	+	28	22	12	92.5	66.7	80.6	85.7	82.2
	-	3	1	24					

+: Positive test result, -: Negative test result Negative: Negative for malignancy PPV:Positive predictive value NPV: Negative predictive value OA: Overall accuracy

The results of UCyt+™ and CK20 expression were compared with urine cytology (Table 1). The sensitivity for the UCyt+™ test was 83.3%, urine cytology 75.9% and CK20 70.3%. The combination of UCyt+™ and cytology, CK20 and cytology, and the three tests together showed sensitivities of 88.9%, 77.7%, and 92.5% respectively.

**Figure 1:**
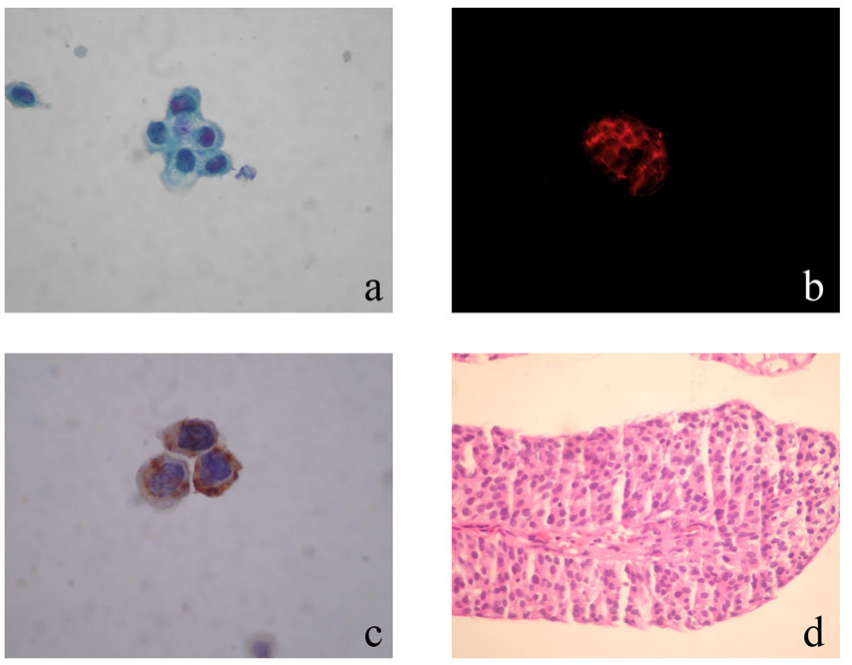
**[A] Low grade urothelial carcinoma cytology (Papanicolaou stain ×200), [B] Positive Immunocyt/uCyt test (red fluorescence × 200), [C] CK20 immunocytochemistry (×200), [D] Biopsy (HE × 200)**.

**Figure 2:**
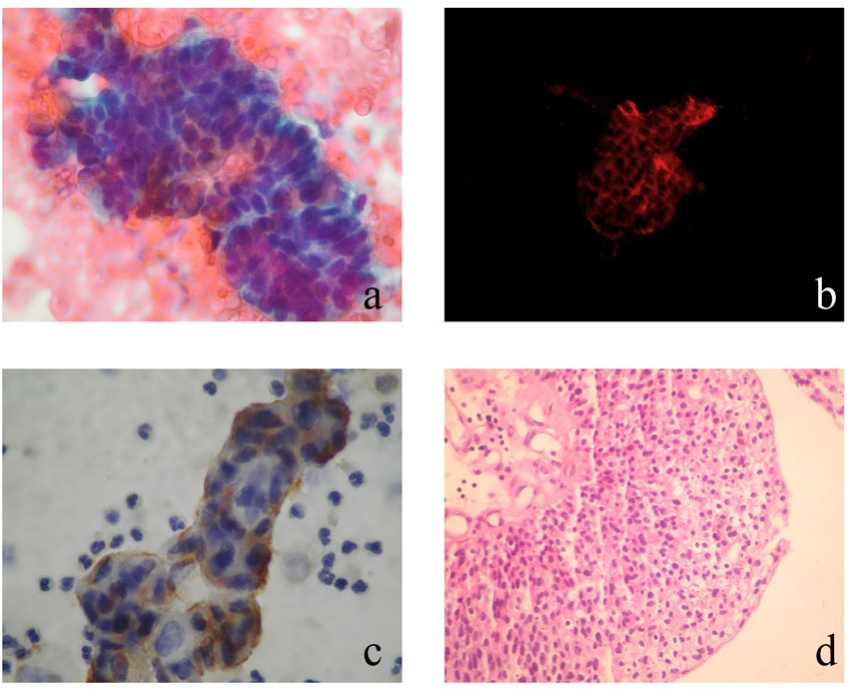
**[A] High grade urothelial carcinoma cytology (Papanicolaou stain × 200), [B] Positive Immunocyt/uCyt test (red fluorescence × 200), [C] CK20 immunocytochemistry (×200), [D] Biopsy (HE × 200)**.

As reported in Table 2, the sensitivity is higher for UCyt+™ and cytology than for CK20 in low grade tumors: 77.4%, 67.7% and 64.5%, respectively (Figure 1). In high grade tumors the sensitivity for UCyt+™ was 91.3%, 86.9% for cytology, and 78.2% for CK20 (Figure 2). The combination of UCyt+™ and cytology had higher sensitivity (83.8%) than the CK20 and cytology combination (70.9%). From the simultaneous evaluation of the three tumor markers, 90.3% of the diagnoses were correct (Table 2).

**Table 2:** Comparison of cytology, ImmunoCyt/uCyt+ and CK20 expression for the detection of different grades of bladder cancer

	**Urothelial tumors**			
	Low grade		High grade	
	
	n = 31	%	n = 23	%

Cytology	21	67.7	20	86.9
UCyt+™	24	77.4	21	91.3
CK20	20	64.5	18	78.2
Cytology+UCyt+™	26	83.8	22	95.6
Cytology+CK20	22	70.9	20	86.9
UCyt+™ +CK20	23	74.1	21	91.3
Cytology+UCyt+™ +CK20	28	90.3	22	95.6

## Discussion

The urine cytology is a useful test in both diagnosis and follow-up and is highly sensitive for detecting high grade tumors; it is limited because of the decreased sensitivity in detecting low-grade tumors. A recent literature review found that the sensitivity of cytology is between 20% to 53%, with a mean of 34%; specificity ranges from 83% to 99.7%, with a mean of 99% [17,18]. Additional screening tests with high sensitivity for tumors of all grades are needed to improve the diagnostic ability of urine cytology and perhaps to reduce the need for frequent cystoscopies, especially in those with low-risk disease.

During the past decade, more than 30 urinary bladder cancer biomarkers have been described [19]. Among these, bladder tumor antigen (BTA Stat and BTA TRAK tests), nuclear matrix protein 22 (NMP-22 enzyme linked immunosorbent assay detection kit) and recently tumor-associated antigens such as M344, 19A211, and LDQ19 (ImmunoCyt fluorescence test), fibrinogen-fibrin degradation products (FDP test), and the UroVysion fluorescent in situ hybridization assay have achieved Food and Drug Administration approval for diagnostic purposes. Still, most of the aforementioned tests are less specific and cost more than conventional cytology [20]. Several large screening studies have demonstrated its low sensitivity and the results of urinary cytology have poor interobserver and intraobserver reproducibility [21].

Halling et al [22] noted that for grade 1, grade 2 and grade 3 bladder tumours, respectively, the grade per grade sensitivity of cytology before 1990 was 37%, 75% and 94% and that it decreased to 11%, 31% and 60% after 1990. The suspected reason for the drop in sensitivity is that, before 1990, studies were conducted by pathologists with great expertise in the field of urine cytology, whereas more recently, cytology has become one of many tests performed by general pathologists lacking direct expertise in urine cytology. In our institute cytologic examination is performed by a trained cytopathologist.

Unlike other urinary markers, UCyt+™ and CK20 are not approved as a stand-alone test but rather, are only approved for use as a surveillance test in conjunction with cytology, which makes direct comparison with other markers more difficult. Overall sensitivity of the combined UCyt+™ and cytology assay has been reported in the range of 81.0%–94.1% [23,24]. Specificity of the combined assay reaches to 61.0%–77.7%, which is less than that offered by cytology alone [24,25]. In our study the sensitivity for cytology is 75.9% and for UCyt+™ is 83.3% and for combination of the both tests are 88.9%. In low grade tumors the sensitivity for UCyt+™ (77.4%) is more than for cytology (67.7%) and for the combination of both tests (83.8%). On the other hand the specificity for cytology (66.7%) reached 86.1% for the combination of the two tests (Table 1). These results are in parallel with most of the reports [11,24].

In our study the CK20 sensitivity is 70.4% and less than for cytology and UCyt+™, but reached 77.8% when combined with urine cytology. Some authors reported sensitivity for CK20 between 86% and 91% with a specificity between 67% and 96% even if in most of the cases the specificity was tested in healthy controls and not in urine from cases of chronic inflammation [25,26]. In these series strong correlation was found between tumor grade and CK20 expression in urine. The sensitivity was higher for CK20 than urine cytology. According to a recently published article the sensitivity of CK20 mRNA was 100% in detecting grade 1 tumors, whereas the sensitivity for high grade (III) was 84.3%. This suggests that CK20 mRNA is a potential tumor marker for the early detection of bladder cancer [27]. In our study we could not find any relation between tumor grade and CK20 expression.

Cytology and cystoscopy have been used as detection tests for patients suspicious for bladder cancer or for the surveillance of patients at risk of tumor recurrence. Cystoscopy is highly sensitive for most tumors but has some practical limitations. It may fail to identify smaller, flat tumors such as carcinoma in situ. Also, despite the technical advances in cystoscopes, the procedure is often perceived as invasive and a source of patient anxiety [28]. There is also a significant financial cost related to frequent cystoscopic monitoring, in terms of health care resources and patient time. Conversely, urinary cytology is noninvasive and highly specific but has poor sensitivity for low-grade, well-differentiated lesions. Thus it cannot be used to replace cystocopy and is used, rather, as an adjunct to help detect occult tumors. Additional screening tests with high sensitivity and specificity for urothelial tumors of all grades are indicated to help improve the diagnostic ability of urine cytology as well as to reduce the need for frequent cystoscopies, especially in those with low-risk disease.

The ImmunoCyt/uCyt+ assay require technical expertise, extensive sample handling and preparation and specialized equipment. However, a person with minimal cytology training and experience can perform the test. The CK20 immunostaining can be used routinely but needs some additional work performed by the technician (bleaching of slides etc).

## Conclusion

In conclusion, combined use of UCyt+™ and cytology can improve the sensitivity and specificity over the CK20 and cytology combination for the detection of bladder cancer in urine. Further new studies with larger patient populations should be done in order to assess the effectiveness of these tests to replace conventional cystoscopy in the primary diagnosis.

## Competing interests

The authors declare that they have no competing interests.

## Authors' contributions

IS participated in the study design, collection of the background references, photomicrography of the immunocytochemical results, displaying the results of the study, writing the discussion of the results, carried out the sequence alignment and drafted the manuscript. FT and FO conceived and designed the study, gave and reviewed the final histopathological diagnosis, and revised the manuscript for important intellectual content. MS, and SS conducted the clinical part of the study and were involved in the design and coordination of the study and drafting the manuscript. All authors read and approved the final manuscript.
